# Three-Basis Loop-Back QKD: A Passive Architecture for Secure and Scalable Quantum Mobile Networks

**DOI:** 10.3390/e27121249

**Published:** 2025-12-11

**Authors:** Luis Adrián Lizama-Pérez, Patricia Morales-Calvo

**Affiliations:** Departamento de Electrónica, Universidad Técnica Federico Santa María, Av. Vicuña Mackenna 3939, San Joaquín, Santiago 7820436, Chile; patricia.morales@usm.cl

**Keywords:** Loop-Back QKD, BB84, quantum communication, quantum mobile networks, error threshold analysis

## Abstract

The Loop-Back Quantum Key Distribution (LB-QKD) protocol establishes a bidirectional architecture in which a single photon travels forth and back through the same optical channel. Unlike conventional one-way schemes such as BB84, Alice performs both state preparation and measurement, while Bob acts as a passive polarization modulator and reflector. This design eliminates detectors at Bob’s side, minimizes synchronization requirements, and enables compact, low-power implementations suitable for quantum-mobile and IoT platforms. An extended three-basis configuration {X,Y,Z} is introduced, preserving the simplicity of the two-basis scheme while improving noise tolerance through enhanced orthogonality-based filtering. Analytical modeling shows that the effective protocol error decreases from $E_{\mathrm{protocol}}^{\left(2\right)}=e/2$ to $E_{\mathrm{protocol}}^{\left(3\right)}=e/3$, achieving a 33% improvement in noise resilience. Despite its slightly lower sifting efficiency (η=1/6), the total information gain reaches G=0.26 bits per pulse, maintaining post-sifting throughput comparable to BB84. The protocol doubles the tolerable QBER of conventional QKD, sustaining secure operation up to 22% for two bases and approximately 47.58% for three bases. Its passive, self-verifying architecture enhances resistance to man-in-the-middle, photon-number-splitting, and side-channel attacks, providing a scalable and energy-efficient framework for secure key distribution and authentication in next-generation mobile and distributed quantum networks.

## 1. Introduction

In the 1970s, Stephen Wiesner proposed a visionary scheme for unforgeable quantum money, based on the fundamental principles of quantum mechanics, particularly the no-cloning theorem [1]. In his proposal, Alice (the bank) encodes quantum states, such as polarizations in orthogonal bases (*X*, *Z*), into a quantum banknote. Bob, the user, presents this banknote, and Alice authenticates its validity by measuring the states in the correct bases, previously stored, thereby detecting any tampering due to the impossibility of cloning unknown quantum states. Although theoretically sound, this concept faced significant technological limitations, as it required quantum memories to preserve the original states—a challenge that was unattainable at the time and remains so today [2].

**Figure 1:**
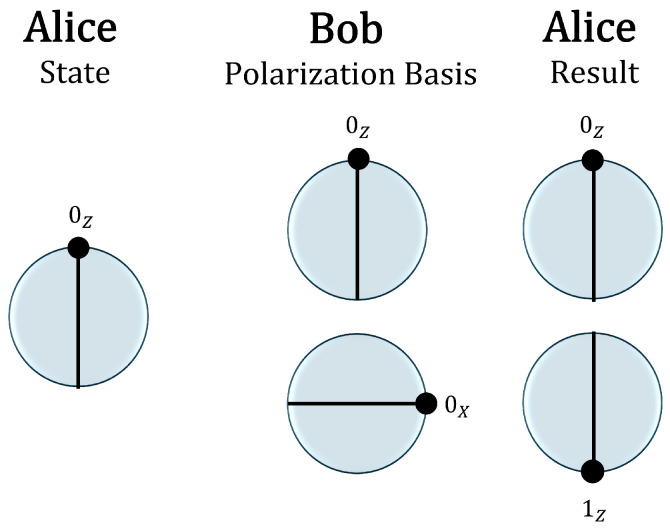
Steps of the protocol: (1) Alice prepares and sends the state $|0_{Z}\rangle$ to Bob using the *Z* basis. (2) Bob randomly selects a basis with equal probability. If he chooses the *Z* basis, the state remains unchanged and is returned to Alice, who measures in the same basis and obtains $|0_{Z}\rangle$. This outcome is ambiguous. (3) If Bob chooses the *X* basis, the state collapses to $|0_{X}\rangle$ or $|1_{X}\rangle$ with equal probability. As illustrated, if the state $|0_{X}\rangle$ is sent back, Alice measures in the *Z* basis and obtains either $|0_{Z}\rangle$ or $|1_{Z}\rangle$ with equal probability. In the latter case, she can infer that Bob used the *X* basis. In this scheme, information is encoded in the basis: Z≡0, X≡1.

The Loop-Back QKD (LB-QKD) protocol realizes this vision by transferring Wiesner’s quantum authentication scheme to an experimentally feasible context [3]. Instead of quantum memories, the polarization bases ($B_{LB}^{\left(j\right)}\in \left\{X,Z\right\}$) are encoded at the receiver (Bob) using an electro-optic modulator (EOM) and a retroreflector. Alice sends attenuated laser pulses with an average photon number μ (typically μ≪1), which Bob reflects after applying a selected basis. Upon reception of the reflected pulse, Alice measures it in the same preparation basis and compares the outcome with the state initially sent. An orthogonal outcome implies that Bob used the conjugate basis, so Alice can infer his basis choice and assign a classical bit value (e.g., Z≡0, X≡1), in direct analogy with Wiesner’s banknote verification scheme illustrated in Figure 1. In this way, each successful round simultaneously authenticates the remote device and contributes one symbol to the raw key. This design removes the need for quantum storage while preserving the essence of the quantum banknote in modern applications. An alternative scenario with static polarizers arranged in a matrix and scanned by Alice further reinforces the analogy with Wiesner’s scheme: each polarizer corresponds to a banknote element, encoded in a fixed basis, whose validity is verified by measurement. The no-cloning principle guarantees security against adversaries. In the dynamic version of LB-QKD, the randomness in basis selection increases flexibility and enables real-time key generation.

**Figure 2:**
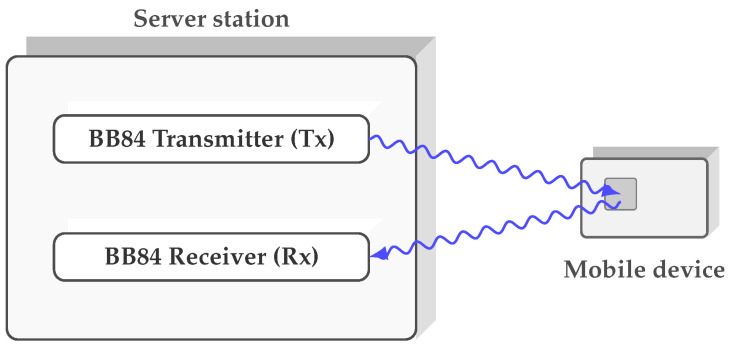
Conceptual diagram of the loop-back architecture. The BB84 transmitter (**Tx**) and receiver (**Rx**) are integrated into a single server station. A mobile device, modeled as a compact token, participates in a quantum loop-back process: a quantum pulse emitted by the **Tx** is directed towards the device, interacts at the mobile interface, and is then returned to the **Rx**, enabling the system either to establish a key with the server or to provide an authentication code.

The BB84 protocol, subsequently developed by Bennett and Brassard, marked a turning point in quantum cryptography by introducing a key distribution method based on the transmission of photons polarized in mutually incompatible bases [4]. Although its security relies on physical principles and has proven robust against both classical and quantum attacks, the protocol requires active components at both ends, precise synchronization, and a complex optical infrastructure. These requirements increase implementation costs and limit integration into portable devices. Traditional one-way DV-QKD protocols such as BB84 require single-photon detection and actively stabilized optics at the receiver, and often also dedicated quantum hardware at the transmitter side. After several decades of development, highly integrated and even commercial implementations of BB84-based QKD are now available, providing turnkey systems for metropolitan and backbone networks [5,6]. However, these architectures typically retain a symmetric allocation of quantum hardware, with single-photon detectors and actively stabilized optics at the receiver and, in many cases, specialized transmitters. In contrast, the Loop-Back architecture concentrates all single-photon detectors and basis-dependent post-processing at a single node (Alice), while the remote station (Bob) can be implemented as a nearly passive device equipped only with a low-power electro-optic modulator and a retroreflector. This asymmetry enables lower-cost and simpler remote terminals and reduces the side-channel exposure at the network edge, which is particularly attractive in scenarios with many distributed users or constrained devices (see Figure 2).

Subsequent extensions, such as B92 [7,8], 6-State [9,10], MDI-QKD [11,12], and TF-QKD [13,14], have aimed to improve efficiency and security, albeit with greater technological demands that hinder widespread deployment.

The driving force behind these advances stems from the development of quantum computing, a disruptive technology capable of solving problems intractable for classical computation. However, the same algorithms that promise to accelerate scientific calculations and process optimization also pose a direct threat to currently used public-key cryptography. Shor’s algorithm, for instance, compromises integer factorization and discrete logarithm computations, which are the foundations of RSA and ECC systems [15,16]. This scenario has motivated the search for solutions offering security grounded in physical principles, such as quantum key distribution (QKD), whose intrinsic nature ensures the detection of any eavesdropping attempts [5,6].

At the international level, significant milestones have been achieved, such as the Beijing–Shanghai quantum network and the Micius satellite mission, which have demonstrated the technical feasibility of large-scale quantum links. Nonetheless, fiber-optic attenuation and the high costs of space-based links remain significant limitations [17,18,19]. In parallel, mobile and portable QKD schemes have been explored, including prototypes developed by Nokia and the University of Oxford, the reference-frame-independent QKD (rfi-QKD) modality, and experimental demonstrations on moving platforms such as drones [20,21,22,23]. These efforts aim to integrate quantum security into everyday applications, although current solutions still face practical constraints.

In this context, the Loop-Back QKD protocol emerges as a particularly attractive alternative. Unlike Wiesner’s quantum banknote, which is unfeasible in its original implementation, and BB84, which requires active infrastructure at both nodes, LB-QKD employs a passive remote device with a low-power electro-optic modulator. The higher technological complexity resides with Alice, who handles transmission, detection, and analysis, while Bob only dynamically selects polarization bases. This design eliminates the need for detectors at Bob’s side, significantly reduces infrastructure requirements, and facilitates device miniaturization. Its analogy to a barcode scanner, where Alice “reads” a passive pattern modulated by Bob, highlights the simplicity and scalability of the approach, positioning it as a viable solution for cybersecurity applications in mobile payments, authentication, and access control in Internet of Things environments.

The remainder of this article is organized as follows. Section 2 describes the Loop-Back QKD protocol and establishes its conceptual and operational equivalence to the standard BB84 scheme. Section 3 introduces the three-basis extension of the Loop-Back protocol, highlighting its structural simplicity and enhanced error tolerance. Section 4 presents a detailed analysis of channel-induced errors and the intrinsic error-compensation mechanisms arising from the round-trip configuration. In Section 5, we derive the analytical bounds for the effective QBER and determine the corresponding security thresholds for both the two- and three-basis configurations. Section 6 discusses the protocol’s robustness against common quantum attacks, including man-in-the-middle, photon-number-splitting, and side-channel exploits. Finally, Section 8 summarizes the main contributions and outlines potential directions for further research and experimental validation.

## 2. The Loop-Back QKD Protocol

The *Loop-Back Quantum Key Distribution* (LB-QKD) protocol establishes a bidirectional quantum communication scheme in which a single optical pulse travels forth and back through the same channel. Unlike one-way protocols such as BB84, the loop-back architecture allows Alice to both prepare and measure the quantum state, while Bob performs only a controlled polarization operation. This configuration inherently verifies the coherence of the optical path and enhances resistance to man-in-the-middle (MitM) attacks, as the quantum channel itself must remain consistent over both directions.

### 2.1. Operational Steps

1. **Parameter Agreement.** Alice and Bob agree on two mutually unbiased polarization bases, $B_{Z}=\left\{|0_{Z}\rangle ,|1_{Z}\rangle \right\}$ and $B_{X}=\left\{|0_{X}\rangle ,|1_{X}\rangle \right\}$, which are associated with logical bits 0 and 1, respectively. They also synchronize timing, detector thresholds, and any classical authentication mechanisms.
2. **State Preparation (Alice).** For each transmission round, Alice randomly selects a preparation basis $B_{A}\in \left\{Z,X\right\}$ and prepares a quantum state $|\psi_{B_{A}}\rangle$ uniformly chosen from $B_{B_{A}}$. The state is then launched into the quantum channel toward Bob.
3. **Random Polarization (Bob).** Upon reception, Bob chooses at random his polarization basis $B_{B}\in \left\{Z,X\right\}$. He applies the corresponding polarization operation to the incoming state and immediately reflects it back to Alice through the same optical path. Bob performs no quantum measurement; his role is purely passive and reflective.
4. **Return and Measurement (Alice).** The pulse travels back through the same optical fiber. Alice measures the returned photon in her original preparation basis $B_{A}$, obtaining one of the two possible outcomes within that basis.
5. **Inference Rule.** If the measurement result is orthogonal to the prepared state, Alice infers that Bob’s basis was opposite to hers ($B_{B}\neq B_{A}$). Conversely, if the measurement coincides with the original state, she concludes that $B_{B}=B_{A}$. Only the former case (orthogonal outcome) is kept for key generation.
6. **Public Announcement and Sifting.** Alice publicly announces only the indices of successful rounds (those with orthogonal outcomes). Bob retains the corresponding rounds in which he used the opposite basis. Both parties now share an implicit bit value determined by Bob’s chosen basis:
  $$B_{B}=Z\Rightarrow \mathrm{bit}\;0,\;B_{B}=X\Rightarrow \mathrm{bit}\;1.$$
7. **Raw Key Formation.** After multiple repetitions of the above procedure, the subset of successful rounds forms the raw key sequence shared between Alice and Bob. Subsequent stages such as error correction, parameter estimation, and privacy amplification are applied later to obtain the final secret key.

The defining feature of LB-QKD is that only Alice performs quantum detection, while Bob’s station acts as a controllable polarization mirror. The correlation between Alice’s measurement outcome and Bob’s random polarization basis allows both parties to share key bits without ever revealing their bases through the public channel. This intrinsic symmetry provides a built-in check of channel integrity and forms the conceptual foundation for advanced loop-back schemes and MitM-resilient network architectures.

### 2.2. BB84-Equivalent Loop-Back

The BB84 protocol constitutes the foundational standard for Quantum Key Distribution (QKD). In its standard version, Alice transmits photons polarized in one of two possible bases, {Z,X}, while Bob measures in a randomly selected basis. Security arises from the fact that an external observer cannot simultaneously know both bases without introducing detectable errors.

**Figure 3:**
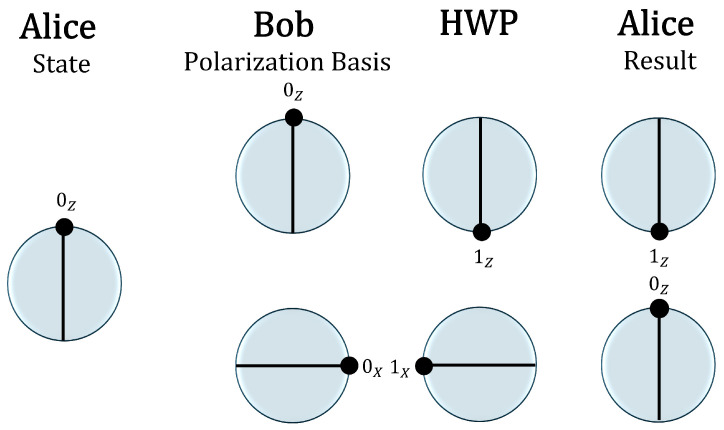
Protocol steps: (1) Alice prepares the state $|0_{Z}\rangle$ and sends it to Bob using the *Z* basis. (2) Bob randomly selects a measurement basis with equal probability. (3) At Alice’s station (fixed Bob), a half-wave plate (HWP) transforms the incoming pulse into the orthogonal quantum state. A successful event occurs when the fixed Bob chooses the same basis as Alice (as in standard BB84) and produces the same measurement outcome, as Alice has access to the results. This constitutes the *Loop-Back equivalent* of the protocol.

In the *BB84-equivalent Loop-Back QKD* protocol, Alice’s station integrates both the transmitter (Tx) and receiver (Rx)—the latter associated with a fixed Bob—and is equipped with a half-wave plate (HWP) (see Figure 3). The mobile Bob, in contrast, only possesses a random polarizer and a dielectric mirror. The pulse is reflected, undergoes a transformation induced by the λ/2 HWP, which effectively returns the orthogonal quantum state, and is subsequently measured by Alice (fixed Bob) in her original preparation basis with a 50% probability.

- BB84 standard: Success occurs when Alice and Bob choose the same basis. The probability of success is 1/2, and after the sifting process, the key is obtained.
- Equivalent Loop-Back: Success occurs when Alice and Bob choose the same basis and, additionally, the measured state matches the state sent by Alice. It is important to note that both the transmitter (Tx) and receiver (Rx) are located at Alice’s station. In this scenario, Alice can infer the basis selected by Bob (mobile). The effective probability of success is 1/4, which is equivalent to the expected yield after discarding events in which the state does not match. Therefore, the adjustment system in the *BB84-equivalent Loop-Back QKD* requires minimal modifications compared to the standard BB84 system.

From an optical viewpoint, the Loop-Back geometry can be interpreted as Alice sending polarized single photons towards a remote module at Bob composed of a polarization-selective element followed by a mirror. For each pulse, Bob selects one of the logical bases {Z,X} and a logical bit by orienting this element, so that the incoming state is projected onto the corresponding polarization mode (with possible loss of the orthogonal component) and then returned along the same path. Operationally, this is equivalent to placing a randomly oriented polarizer on the round-trip path in front of a mirror, with the distinctive feature that all photodetection takes place locally at Alice, while Bob’s module only implements polarization selection before reflection.

It should be noted that the component described as a dielectric mirror in Bob’s configuration specifically refers to a *retroreflector* (e.g., a corner-cube). This device reflects quantum pulses back along the direction of incidence regardless of Bob’s angular orientation, which is critical for mobile applications where the receiver device (e.g., tag, smartphone, or IoT device) may change position or orientation during transmission.

A key advantage of the Loop-Back QKD scheme is that Bob’s hardware operates in an almost passive manner, as it does not require an active pulse detection system. This also provides another significant benefit: Alice and Bob do not need complex synchronization, since Alice’s objective is to identify the basis $B_{LB}\in \left\{X,Z\right\}$ used by Bob at the detection instants.

## 3. The Three-Basis Loop-Back QKD Protocol

The *Three-Basis Loop-Back Quantum Key Distribution* (LB-QKD) protocol extends the bidirectional configuration of the original two-basis scheme by operating over three mutually orthogonal polarization bases, $B_{X}=\left\{|0_{X}\rangle ,|1_{X}\rangle \right\}$, $B_{Y}=\left\{|0_{Y}\rangle ,|1_{Y}\rangle \right\}$, and $B_{Z}=\left\{|0_{Z}\rangle ,|1_{Z}\rangle \right\}$. The system preserves the core principle of the Loop-Back architecture: Alice performs both state preparation and measurement, while Bob only applies a polarization transformation and reflects the photon without detection. This configuration maintains bidirectional coherence and provides enhanced error filtering due to the triaxial encoding.

### Operational Steps

1. **Parameter Agreement.** Alice and Bob agree on the three mutually unbiased polarization bases {X,Y,Z}, each associated with a distinct logical label:
  X→00,Y→01,Z→10.
  They synchronize timing, detector thresholds, and classical authentication parameters as in the two-basis configuration.
2. **State Preparation (Alice).** In each transmission round, Alice randomly selects a preparation basis $B_{A}\in \left\{X,Y,Z\right\}$ and prepares a quantum state $|\psi_{B_{A}}\rangle$ uniformly chosen from the corresponding basis $B_{B_{A}}$. The photon is then sent through the quantum channel to Bob.
3. **Random Polarization (Bob).** Upon reception, Bob randomly selects his polarization basis $B_{B}\in \left\{X,Y,Z\right\}$ with uniform probability 1/3. He applies the corresponding polarization transformation to the incoming state and immediately reflects it back toward Alice through the same optical path. Bob performs no quantum measurement; his role remains purely passive.
4. **Return and Measurement (Alice).** The photon travels back through the same optical fiber. Alice measures it in her original basis $B_{A}$, obtaining one of the two possible outcomes in that basis.
5. **Inference Rule.** If the measurement result is orthogonal to the state she initially sent, Alice infers that Bob’s basis was different from hers ($B_{B}\neq B_{A}$). In this case, Bob’s chosen basis must belong to the plane orthogonal to her own preparation basis.
6. **Public Announcement and Sifting.** To enable basis identification without revealing it directly, Bob publicly announces a pair of bases selected from {XY,XZ,YZ}, ensuring that his actual basis is one of the two in the announced pair. Among these three possible pairs, exactly one does not include Alice’s basis and is therefore ambiguous. If the announced pair excludes $B_{A}$, the round is discarded. If the announced pair includes $B_{A}$ and Alice’s measurement result was orthogonal, the round is retained for key generation, as Alice can unambiguously determine Bob’s basis.
7. **Raw Key Formation.** The retained rounds form the raw key, with bit values assigned according to Bob’s basis using the mapping
  X→00,Y→01,Z→10.
  Subsequent stages of error correction and privacy amplification are applied to distill the final secret key.

In ideal conditions, the probability that Bob selects a basis different from Alice’s is 2/3, the probability that the announced pair includes Alice’s basis is 1/2, and the probability of obtaining an orthogonal outcome in Alice’s measurement is 1/2. The resulting sifting efficiency is therefore

$$\eta =\frac{2}{3}\times \frac{1}{2}\times \frac{1}{2}=\frac{1}{6}\approx 16.7\%.$$

Given that three bases convey $\log_{2}\left(3\right)=1.585$ bits of information, the theoretical information gain is

$$G=\eta \log_{2}\left(3\right)\approx 0.26\;\mathrm{bits}\;\mathrm{per}\;\mathrm{pulse}.$$

This scheme maintains a comparable efficiency to the two-basis version while achieving a slightly higher information gain of approximately 0.26 bits per pulse.

## 4. Channel Error Analysis

We now extend the analysis of the quantum bit error rate (QBER) to compare the two- and three-basis versions of the Loop-Back QKD protocol. Let *e* denote the error probability introduced by the quantum channel during one-way propagation. The Loop-Back architecture allows partial compensation of these errors through Bob’s active polarization regeneration and Alice’s orthogonality-based filtering.

### 4.1. Two-Basis Configuration

Consider first the bidimensional configuration operating over the bases {X,Z}. When Alice and Bob select the same basis, the polarization transformation applied by Bob acts as an active regenerator, partially compensating for the forward-path distortion. Under this condition, the effective error seen by Alice is approximately equal to the channel error,

$$E_{\mathrm{same}}^{\left(2\right)}\approx e.$$

When the bases differ ($B_{A}\neq B_{B}$), Alice measures the returned photon in her preparation basis. The projection and orthogonality acceptance criterion remove most of the depolarization effects, leading to

$$E_{\mathrm{different}}^{\left(2\right)}=0.$$

Since each basis is chosen with equal probability, the overall average error over the accepted rounds is

$$E_{\mathrm{protocol}}^{\left(2\right)}=\frac{1}{2}E_{\mathrm{same}}^{\left(2\right)}+\frac{1}{2}E_{\mathrm{different}}^{\left(2\right)}=\frac{e}{2}.$$

This error reduction by a factor of two relative to the raw channel error explains the improved tolerance and longer operational distance compared to BB84, while preserving a comparable key rate.

### 4.2. Three-Basis Configuration

In the triaxial configuration using {X,Y,Z}, the probabilities of coinciding and differing bases change: the chance of selecting the same basis becomes 1/3, while differing bases occur with probability 2/3. As before, only the same-basis cases accumulate the full channel error, while the mismatched rounds are largely immune to depolarization due to the filtering mechanism:

$$E_{\mathrm{same}}^{\left(3\right)}\approx e,\;E_{\mathrm{different}}^{\left(3\right)}=0.$$

Therefore, the average protocol error for the three-basis system becomes

$$E_{\mathrm{protocol}}^{\left(3\right)}=\frac{1}{3}E_{\mathrm{same}}^{\left(3\right)}+\frac{2}{3}E_{\mathrm{different}}^{\left(3\right)}=\frac{e}{3}.$$

The expected QBER of the accepted qubits is thus further reduced by a factor of 1/3, yielding an effective improvement in noise resilience compared to the two-basis scheme. This property implies that the secret key rate remains positive under higher physical QBER or longer transmission distances.

### 4.3. Comparative Discussion

While the sifting efficiency of the three-basis scheme is slightly lower ($\eta_{3B}=1/6\approx 16.7\%$) than that of the two-basis configuration ($\eta_{2B}=1/4=25\%$), the overall information gain remains comparable:

$$G_{2B}=\eta_{2B}\log_{2}\left(2\right)=0.25\;\mathrm{bits}/\mathrm{pulse},\;G_{3B}=\eta_{3B}\log_{2}\left(3\right)\approx 0.26\;\mathrm{bits}/\mathrm{pulse}.$$

Hence, the advantage of the three-basis protocol lies not in the information throughput but in its intrinsic robustness to channel errors. The effective error ratio,

$$\frac{E_{\mathrm{protocol}}^{\left(3\right)}}{E_{\mathrm{protocol}}^{\left(2\right)}}=\frac{\left(e/3\right)}{\left(e/2\right)}=\frac{2}{3},$$

quantifies a relative 33% improvement in error suppression over the two-basis case.

## 5. Error Thresholds

The asymptotic secret-key rate *R* for the Loop-Back QKD protocol is evaluated using the Devetak–Winter bound under the assumption of collective symmetric attacks. In this idealized framework, *R* depends on the mutual information I(A:B) shared between Alice and Bob and on the Holevo bound χ(A:E) representing Eve’s accessible information. Here, collective symmetric attacks refer to the standard scenario in which Eve applies the same interaction to each signal, stores her quantum ancillae in a long-term quantum memory and performs a joint measurement at the end of the protocol. The Devetak–Winter bound then provides a lower bound on the asymptotic secret-key rate under this restricted but widely used attack model. A rigorous extension of our analysis to the most general coherent attacks in two-way architectures such as Loop-Back QKD, where Eve may correlate arbitrarily many rounds at the level of the global quantum state, is beyond the scope of this work and remains an open problem. Therefore, the error thresholds derived in Section 5 should be interpreted as asymptotic benchmarks under symmetric collective attacks, rather than as full-security bounds against all possible coherent strategies.

Within this framework, the action of the physical quantum channel on a single polarization qubit can be represented by the isotropic depolarizing map

(1) $$E_{e}\left(\rho \right)=\left(1-e\right)\;\rho +e\;\frac{I_{2}}{2},$$

where *e* denotes the one-way depolarizing probability. Here $I_{2}$ stands for the 2×2 identity operator on the polarization qubit, so that $I_{2}/2$ is the maximally mixed state. In other words, with probability 1−e the channel leaves the state ρ unchanged, while with probability *e* it replaces it by uniform white noise on the Bloch sphere. For symmetric collective attacks this depolarizing model reproduces the observed QBER in the honest implementation and provides an effective description of Eve’s optimal interaction with each signal.

For symmetric channels, these quantities can be expressed as

$$I\left(A:B\right)=\log_{2}n-H\left(B|A\right),\;\chi \left(A:E\right)\approx H\left(B|A\right),$$

where *n* denotes the number of polarization bases defining the protocol (two or three), and H(B|A) is the conditional Shannon entropy of Bob’s basis given Alice’s preparation. In the Loop-Back setting, this symmetry arises from two ingredients. First, Alice and Bob choose each of the *n* mutually unbiased bases with equal probability, so that all logical symbols are used in a perfectly balanced way. Second, the physical channel is modeled as an isotropic depolarizing map acting on the polarization qubit, which affects all basis states in the same way. As a consequence, the effective classical channel from Alice’s basis label *A* to Bob’s reconstructed label *B* is a discrete symmetric channel: for any input symbol *a*, the correct output occurs with probability 1−E, while the n−1 error symbols occur with equal probability E/(n−1).

The resulting asymptotic key rate is

(2) $$R\geq \eta \left[\log_{2}n-2H\left(B|A\right)\right],$$

with η being the sifting efficiency.

### 5.1. Two-Basis Configuration

For the standard Loop-Back QKD protocol with two bases (n=2, {X,Z}), the quantum bit error rate (QBER) in the accepted key is denoted by E=e/2, where *e* is the one-way physical channel error. In what follows, we model this physical error as arising from an isotropic depolarizing channel acting on the polarization qubit. In this picture, *e* plays the role of the depolarizing probability per one-way use of the link, effectively capturing the aggregate effect of random polarization rotations and decoherence in the optical channel. In this case, H(B|A) reduces to the binary entropy function:

$$H\left(B|A\right)=h\left(E\right)=-E\log_{2}E-\left(1-E\right)\log_{2}\left(1-E\right).$$

Setting R=0 in Equation (2) gives the critical condition

$$\log_{2}2=2h\left(E_{\mathrm{th}}\right)\;\;\Rightarrow \;\;h\left(E_{\mathrm{th}}\right)=\frac{1}{2}.$$

Solving numerically yields $E_{\mathrm{th}}\approx 0.11$. Using E=e/2, the maximum tolerable physical error is

$$e_{\mathrm{th}}^{\left(2\right)}=2E_{\mathrm{th}}\approx 0.22.$$

Hence, the two-basis Loop-Back protocol doubles the tolerable physical QBER compared to the standard BB84 threshold ($e_{\mathrm{BB}84}\approx 0.11$), as a result of bidirectional propagation and orthogonality-based filtering.

### 5.2. Three-Basis Configuration

For the extended Loop-Back QKD protocol employing three orthogonal polarization bases (n=3, {X,Y,Z}), the effective QBER is reduced to E=e/3 following the Devetak–Winter bound for symmetric collective attacks [24]. Here, Bob’s outcomes form a ternary symmetric channel, conceptually related to the three-basis (six-state) model introduced by Bruß [9] with correct transmission probability (1−E) and two equiprobable error outcomes each of probability E/2. This structure follows directly from the combination of uniform basis selection over {X,Y,Z} and isotropic depolarization: conditioned on Alice’s basis label *A*, Bob obtains the same label with probability 1−E, while each of the two remaining labels occurs with probability E/2, defining a standard ternary symmetric channel. The conditional entropy becomes

$$H\left(B|A\right)=-\left(1-E\right)\log_{2}\left(1-E\right)-E\log_{2}\;\left(\frac{E}{2}\right).$$

Substituting this into Equation (2) and setting R=0 gives the threshold condition

$$\log_{2}3=2H\left(B|A_{\mathrm{th}}\right),$$

which corresponds to $H(B|A_{\mathrm{th}})=0.7925$. Solving numerically yields $E_{\mathrm{th}}\approx 0.1595$, and therefore

$$e_{\mathrm{th}}^{\left(3\right)}=3E_{\mathrm{th}}\approx 0.4785.$$

This represents an idealized upper bound under perfect ternary symmetry: in this regime, the effective error in the accepted key (E=0.1595) remains below the Shannon limit where the mutual information vanishes.

The binary and ternary derivations provide complementary perspectives on the Loop-Back protocol’s error tolerance. The two-basis formulation, employing binary entropy h(E), yields the effective threshold $E_{\mathrm{th}}^{\left(2\right)}$ and the corresponding physical limit $e_{\mathrm{th}}^{\left(2\right)}\approx 0.22$, which is twice that of BB84. The three-basis model, based on ternary entropy H(B|A), yields the higher bound $e_{\mathrm{th}}^{\left(3\right)}\approx 0.48$, emphasizing the robustness of triaxial encoding under isotropic depolarization.

It is important to stress the scope and limitations of this ternary-entropy threshold. The use of the ternary Shannon entropy in the three-basis Loop-Back configuration relies on modelling the sifted key as a discrete symmetric channel with a three-symbol logical alphabet, in which the correct symbol is obtained with probability $1-E_{\mathrm{protocol}}$ and each of the two error symbols occurs with equal probability $E_{\mathrm{protocol}}/2$. This structure is formally analogous to the one encountered in the six-state protocol under symmetric depolarizing noise and collective attacks, where three mutually unbiased bases are used and the resulting bit and phase errors are treated on equal footing.

However, the Loop-Back architecture is not equivalent to the standard six-state scheme [9]. In particular, Loop-Back QKD employs a two-way, round-trip geometry with orthogonality-based filtering at Alice’s station, and Bob plays a strictly passive role without local measurements or active encoding of key bits. As a consequence, existing unconditional security proofs and error thresholds derived for the six-state protocol cannot be directly applied to the Loop-Back setting. The values $e_{\mathrm{th}}^{\left(2\right)}$ and $e_{\mathrm{th}}^{\left(3\right)}$ reported here should therefore be understood as asymptotic performance benchmarks within the isotropic depolarizing-channel model and under symmetric collective attacks, rather than as rigorous six-state security bounds applied verbatim to the Loop-Back protocol.

Moreover, our analysis neglects finite-size effects, possible asymmetries between the error rates associated with different bases, and implementation-specific imperfections beyond their contribution to the overall QBER. These factors may shift the practical thresholds in real devices, and a full treatment of finite-key corrections and device-level parameters is left for future work.

The use of an isotropic depolarizing channel as an effective description of polarization noise is standard in QKD performance and security studies, where the various physical imperfections of the optical link are lumped into a single depolarizing parameter that reproduces the observed QBER and bounds the impact of eavesdropping strategies [25,26]. For polarization-encoded implementations, random birefringence fluctuations, misalignment and environmental perturbations tend to scramble the state of polarization over time; the time-averaged action of these effects is well captured by an isotropic depolarizing map that shrinks the Bloch vector toward the maximally mixed state [27,28]. Within this framework, the parameter *e* quantifies the depolarizing probability per one-way channel use, while additional implementation-dependent imperfections (such as detector dark counts or timing jitter) can be treated as extra contributions to the QBER that affect Loop-Back and one-way protocols in a comparable way, without altering the relative robustness indicated by $e_{\mathrm{th}}^{\left(2\right)}$ and $e_{\mathrm{th}}^{\left(3\right)}$.

In practice, real optical channels are neither perfectly symmetric nor memoryless, so the effective threshold will lie between these two limits. Nevertheless, the ternary extension highlights the key advantage of Loop-Back QKD: by expanding the Hilbert space dimensionality and employing bidirectional verification, the protocol distributes channel noise over more degrees of freedom, effectively increasing the tolerable physical error while preserving secure key generation.

**Figure 4:**
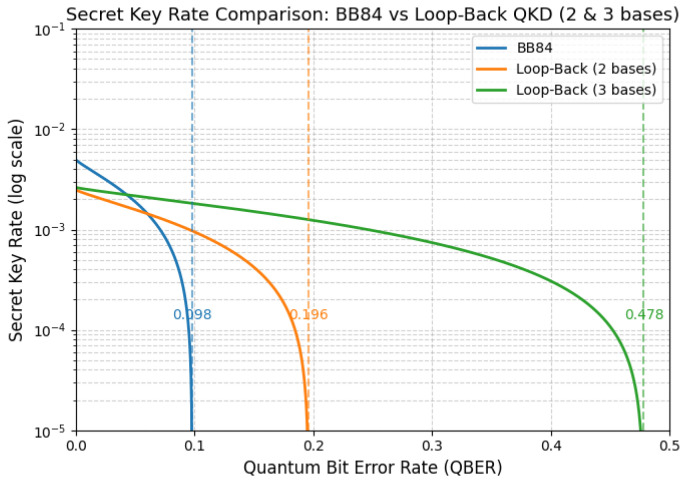
Comparison of the effective quantum bit error rate (QBER) and the corresponding secret key rate for the two- and three-basis Loop-Back QKD configurations. The three-basis scheme exhibits a reduced effective protocol error ($E_{\mathrm{protocol}}^{\left(3\right)}=e/3$) and maintains a positive secret key rate under higher-channel-noise conditions.

The combined effect of this reduction is a slower decay of the secret key rate as the QBER increases, as shown in Figure 4. The three-basis scheme maintains positive key generation at higher channel noise levels and extends the secure communication distance without requiring additional optical components. This behavior stems from the expansion of the polarization space, which increases the proportion of rounds that benefit from orthogonality-based filtering.

## 6. Security Analysis

The Loop-Back QKD protocol belongs to the class of bidirectional quantum communication schemes, where the same photon traverses the channel twice—first from Alice to Bob and then back to Alice. This topology establishes a natural connection with earlier deterministic communication protocols, notably the LM05 scheme proposed by Lucamarini and Mancini in 2005 [29]. While both architectures exploit the round-trip propagation of a single photon, their objectives, encoding strategies, and physical security assumptions differ fundamentally.

### 6.1. Operational and Conceptual Paradigm

In the LM05 protocol, the photon acts as a deterministic carrier of information. Alice prepares a qubit in one of two nonorthogonal bases (*Z* or *X*) and transmits it to Bob, who encodes his bit by applying one of two unitary operations,

$$U_{0}=I,\;U_{1}=iY.$$

The unitary iY performs a π rotation on the Bloch sphere, mapping each logical state to its orthogonal complement in both bases. After applying the transformation, Bob returns the photon to Alice, who measures it in the same basis used for preparation. This configuration achieves deterministic communication: in the absence of noise, Alice can infer Bob’s bit value with certainty, without discarding events or performing basis reconciliation.

Loop-Back QKD, in contrast, remains strictly probabilistic. Bob does not encode any information; instead, he randomly polarizes the incoming photon in one of the allowed bases and reflects it. All bit generation and basis inference occur at Alice’s side, using only the subset of orthogonal measurement outcomes. This design choice preserves the randomness necessary for secure key distribution and shifts the focus from deterministic information transfer to entropy extraction and continuous channel verification.

### 6.2. Security Implications

The structural divergence between LM05 and Loop-Back QKD yields distinct security profiles. In LM05, Bob’s active unitary modulation requires electro-optic devices synchronized to Alice’s emission, introducing an optical activity that becomes a potential source of side-channel leakage. Residual back-reflections or phase fluctuations may carry information about the applied operation, enabling Trojan-Horse Attacks (THA) [30], in which an eavesdropper injects probe light to infer Bob’s modulator state. Although countermeasures such as optical isolators, power monitors, and authentication layers can mitigate these attacks, they increase the system’s hardware complexity and synchronization requirements.

Loop-Back QKD eliminates the need for active modulation altogether. Bob performs no unitary encoding, measurement, or detection. His setup consists of a passive polarization modulator operating under a random control signal and a retroreflector that ensures spatial mode reversibility. No data is stored or processed in Bob’s node, making the system inherently immune to Trojan-Horse probing: an injected photon cannot interact with any basis-dependent optical component capable of encoding useful information. Furthermore, the absence of emissions or switching transients suppresses classical side-channel leakage, including electromagnetic or thermal signatures. The protocol thus achieves higher security not through added hardware, but through architectural minimalism—a reduction in attack surface by design.

### 6.3. Intrinsic Security Properties of the Loop-Back Protocol

Beyond its structural resilience, Loop-Back QKD integrates continuous channel verification within its operation. Every photon traverses the quantum link twice, and deviations in the expected statistics of orthogonal detections immediately reveal channel perturbations or interception. This built-in monitoring replaces the *control mode* used in LM05, where Bob alternates between coding and reflection to estimate the QBER. Loop-Back achieves the same verification intrinsically, without mode switching or classical disclosure.

Resistance to Man-in-the-Middle (MiTM) Attacks

As previously discussed in [3], the round-trip propagation in Loop-Back QKD allows Alice to verify channel integrity by monitoring unmodified reflections. Any deviation from the expected error rate serves as a direct indicator of eavesdropping. Moreover, an adversary cannot conceal the temporal delay Δt introduced by an intermediate relay. Even minimal tampering produces a cumulative delay of 2Δt over the ABA path. Precise round-trip timing analysis thus enables physical-layer detection of MitM attempts without requiring additional quantum resources.

To make this point more explicit in the three-basis configuration, let us consider the case in which Alice prepares the state $|0_{Z}\rangle$ and always measures in the *Z* basis. We denote by $p_{⊥}$ the probability that Alice obtains the orthogonal outcome $|1_{Z}\rangle$, which is precisely the subset of events retained by the orthogonality-based filtering stage. In the honest implementation, Bob chooses his basis $B_{B}\in \left\{X,Y,Z\right\}$ uniformly at random. A successful orthogonal event can only occur when Bob uses one of the two complementary bases *X* or *Y*, and in that case the returning photon has equal overlap with $|0_{Z}\rangle$ and $|1_{Z}\rangle$. The probability of an orthogonal outcome in the absence of an eavesdropper is therefore

(3) $$p_{⊥}^{\left(0\right)}=\mathrm{Pr}\left(B_{B}\in \left\{X,Y\right\}\right)\;\mathrm{Pr}\left(1_{Z}|B_{B}\in \left\{X,Y\right\}\right)=\frac{2}{3}\times \frac{1}{2}=\frac{1}{3}.$$

Now suppose that Eve inserts a station identical to Alice’s and performs a two-way intercept–resend attack. She measures the forward photon in a randomly chosen basis $B_{E}\in \left\{X,Y,Z\right\}$, resends the detected state to Bob, intercepts the reflected photon again in the same basis $B_{E}$, and finally prepares the corresponding state towards Alice. If Eve chooses the correct basis $B_{E}=Z$ (with probability 1/3), her intervention is transparent and the protocol statistics remain unchanged,

(4) $$p_{⊥}^{\left(Z\right)}=\frac{1}{3}.$$

In contrast, when Eve chooses one of the complementary bases *X* or *Y* (total probability 2/3), her double measurement–preparation stage modifies the interference pattern of the round-trip photon. A branch-by-branch analysis of this case (summarized in Table A1 in Appendix A, for the representative input $|0_{Z}\rangle$ and Eve’s forward outcome $|0_{X}\rangle$) shows that Alice obtains the orthogonal outcome $|1_{Z}\rangle$ with probability $p_{⊥}^{\left(X/Y\right)}=5/6$.

Averaging over Eve’s random choice of basis we obtain the overall orthogonal-outcome probability in the presence of this man-in-the-middle attack,

(5) $$p_{⊥}^{\left(\mathrm{Eve}\right)}=\mathrm{Pr}\left(B_{E}=Z\right)\;p_{⊥}^{\left(Z\right)}+\mathrm{Pr}\left(B_{E}=X\;\mathrm{or}\;Y\right)\;p_{⊥}^{\left(X/Y\right)}=\frac{1}{3}\cdot \frac{1}{3}+\frac{2}{3}\cdot \frac{5}{6}=\frac{2}{3}.$$

Thus, when Eve attempts to impersonate Bob in this way, the frequency of orthogonal events at Alice’s station doubles from $p_{⊥}^{\left(0\right)}=1/3$ to $p_{⊥}^{\left(\mathrm{Eve}\right)}=2/3$. Together with the corresponding increase in the QBER of the sifted data, this macroscopic deviation from the expected statistics provides a clear signature of the attack during the parameter-estimation stage.

Resistance to Photon Number Splitting (PNS) Attacks

Photon-number-splitting attacks exploit multi-photon emissions to gain partial information about the key. In Loop-Back QKD, Bob’s passive configuration—using a polarization modulator and retroreflector without detectors—prevents any measurement leakage from Bob’s side. Since the polarization bases are never disclosed over the classical channel and are inferred solely by Alice through successful detection events ($|\xi^{\left(i\right)}\rangle =|\psi^{\left(i\right)}\rangle$), Eve cannot correlate her intercepted photons with Bob’s actual basis choices.

Protection Against Side-Channel Attacks

Side-channel vulnerabilities, such as power consumption analysis or electromagnetic emissions, are inherently suppressed by the passive nature of Bob’s module. In most DV-QKD implementations, the most technologically demanding and attack-prone components are the single-photon detectors at the receiver side, whose efficiency, dark-count rate and afterpulsing behavior directly limit the achievable key rate and enable powerful detector side-channel attacks [5,31]. By design, the Loop-Back architecture removes all detection hardware from the remote node and concentrates it at Alice’s station, so that Bob does not include single-photon detectors, time-tagging electronics or local key-dependent processing. Compared with other two-way QKD schemes where the remote node plays a nominally passive role (such as LM05- and Ping-Pong-like protocols, or plug-and-play architectures that still rely on local detectors, entangled sources or active encoders at the remote site) [29,32,33], the Loop-Back configuration keeps all basis-dependent detection and post-processing at Alice. The remote device only performs basis selection via a low-power electro-optic modulator and a retroreflector, and never stores or measures key-dependent information, which provides a concrete security advantage by further reducing the available attack surface at Bob’s side. The electro-optic modulator operates at constant low power, producing no deterministic timing pattern related to the chosen basis. Furthermore, the non-disclosure of bases in classical communication eliminates the possibility of statistical correlation with external measurements, thereby minimizing leakage channels such as EOM switching times or synchronization artifacts.

Regarding Trojan-horse strategies, we consider the usual scenario in which Eve injects bright or faint coherent pulses into Bob’s optical port in order to gain information about the internal setting of the polarization modulator. In the Loop-Back architecture, such information would reveal Bob’s basis choice but not any locally stored key bits, since Bob does not perform measurements, does not encode classical data, and does not keep secret-dependent records. We assume that standard countermeasures are implemented at Bob’s module, such as optical isolators, narrowband filtering, power monitoring, and randomized attenuation, so that any residual information leakage can be bounded and incorporated into the effective QBER parameter used in our security analysis. A detailed device-level modelling of Trojan-horse attacks for specific hardware realizations of Loop-Back QKD is left for future work, and the main implications for the present analysis are summarized in Table 1.

Limitations and Practical Considerations

Despite the above advantages, the Loop-Back architecture also presents some intrinsic limitations. The most relevant one is its sifting efficiency: in the current two- and three-basis configurations, only about 25% and 26% of the detected rounds, respectively, contribute to the raw key, in contrast with the 50% basis-matching efficiency of standard BB84. From a purely rate-oriented perspective, this represents a disadvantage with respect to optimized one-way DV-QKD schemes when operated at comparable clock frequencies. However, in short-range applications such as device authentication, access-control or payment terminals, the required amount of secret key per session is modest and the logical authentication string can be embedded into a redundant, shuffled encoding, so that only a subset of the physical positions must be successfully received to reconstruct the underlying code. In this way, the reduced sifting efficiency is addressed at the protocol level using simple classical redundancy, without demanding more aggressive quantum hardware.

Interestingly, the main quantitative advantage of Loop-Back lies in its robustness against isotropic depolarizing noise, which is typically associated with long-distance or highly perturbed optical channels. This feature can nevertheless be exploited in short-range but non-ideal environments, for instance when the remote device is partially occluded or embedded in a non-transparent medium (e.g., inside bags, cases or enclosures) that introduces strong polarization scrambling. In such settings, the enhanced depolarization tolerance becomes a relevant asset compared with one-way protocols using the same hardware resources.

Finally, the security analysis presented in this work is carried out under an idealized symmetric depolarizing-channel model and in the asymptotic regime. Extending the treatment to include finite-size effects, realistic device imperfections and more general attack models is an important direction for future work. Moreover, as in any bidirectional QKD protocol, the Loop-Back configuration requires standard countermeasures against bright-light and Trojan-horse attacks on the remote module, even though the absence of single-photon detectors at Bob already reduces his side-channel exposure.

A concise overview of the error models and attack scenarios considered in this work, together with their implications for the Loop-Back architecture, is presented in Table 1.

## 7. Advances and Perspectives

The Loop-Back QKD architecture represents a structural evolution of bidirectional quantum communication protocols such as LM05, achieving passive operation, intrinsic channel verification, and continuous intrusion detection. Building upon this foundation, the introduction of a three-basis configuration extends the theoretical robustness of the system, improving its tolerance to depolarization and its statistical stability without increasing experimental complexity.

The transition from LM05 to Loop-Back QKD replaces active modulation with passive reflection, transforming Bob from an information-encoding node into a purely optical regenerator. This structural simplification removes side-channel vulnerabilities while preserving the compensating effect of bidirectional propagation. The next logical step—the extension from two to three polarization bases—constitutes a dimensional expansion of the encoding space. While the average sifting efficiency slightly decreases to $\eta_{3B}=1/6$, the protocol benefits from a lower effective error rate and greater noise resilience.

In the two-basis configuration, half of the transmitted rounds correspond to matching bases, which fully experience the channel error *e*, while the remaining half benefit from the orthogonality-based filtering that effectively cancels depolarization. In the three-basis system, the probability of matching bases is reduced to 1/3, and two-thirds of the rounds exploit the filtering effect. As a result, the effective error rate for the accepted key bits becomes

$$E_{\mathrm{protocol}}^{\left(3\right)}=\frac{1}{3}e,$$

compared to $E_{\mathrm{protocol}}^{\left(2\right)}=e/2$ for the two-basis case. This reduction by one third translates into improved noise tolerance and a more gradual degradation of the secret key rate as the channel QBER increases.

Beyond error suppression, the use of three mutually orthogonal bases introduces statistical symmetry into the operation of the protocol. In the two-basis version, the basis pairings alternate between *X* and *Z*, producing an inherent anisotropy in the sampling of polarization states. The triaxial configuration uniformly distributes state preparation and reflection across the Bloch sphere, reducing systematic bias and improving the homogeneity of the raw key distribution. This property strengthens the resistance to adaptive attacks and polarization drift, particularly over long-distance fiber links where birefringence fluctuates dynamically.

Moreover, the three-basis framework generalizes the principle of orthogonality-based inference: since each measurement plane shares overlap with the other two, every mismatched event contributes indirectly to the estimation of channel stability. This continuous sampling of orthogonal subspaces enhances real-time monitoring and allows the system to distinguish depolarization effects from deliberate eavesdropping with higher statistical confidence.

The implementation of the three-basis Loop-Back protocol does not require additional optical components beyond those used in the two-basis version. The same electro-optic modulator (EOM) and retroreflector can be operated under three discrete voltage settings corresponding to the bases *X*, *Y*, and *Z*. Synchronization and timing constraints remain identical, as all modulation events are passive and randomized. Consequently, the scalability of the system is achieved entirely in software—through an expanded control sequence—without modification of the optical hardware.

From a practical perspective, the three-basis Loop-Back configuration combines the advantages of passive bidirectional QKD with an expanded polarization space that provides improved noise resilience and enhanced statistical uniformity. Although the raw information gain remains comparable to the two-basis version ($G_{3B}\approx 0.26$ bits/pulse), the reduction in effective channel error ($E_{\mathrm{protocol}}^{\left(3\right)}=e/3$) directly translates into an extended secure communication range and increased stability under fluctuating channel conditions.

In the context of short-range deployments, an interesting direction for future work is the integration of Loop-Back QKD with tailored classical coding for authentication. Instead of requiring every quantum round to contribute directly to a raw key, the logical authentication string could be embedded into a redundant, shuffled pattern spread over multiple Loop-Back exchanges, so that only a subset of the detected positions needs to be successfully received to reconstruct the underlying code. This approach shifts part of the burden from quantum hardware to classical post-processing, leveraging simple repetition- or erasure-like coding combined with random interleaving. A full information-theoretic and implementation-level study of such redundancy schemes, including optimal overhead, robustness against noise and joint security with the quantum layer, is left for future work.

## 8. Conclusions

The Loop-Back QKD protocol provides a versatile framework for secure key distribution and authentication across scalable and mobile quantum networks, balancing efficiency, security, and implementation simplicity. Its quasi-passive Bob architecture—requiring minimal hardware and no detection modules—makes it particularly suitable for low-power or space-constrained environments such as portable quantum devices, edge nodes, and IoT infrastructures. By concentrating all active functionality at Alice’s station, the system supports compact, energy-efficient deployments with reduced synchronization and calibration overhead.

A principal theoretical advantage of the scheme lies in its intrinsic error-suppression mechanism: the combination of active polarization regeneration and orthogonality-based filtering reduces the effective channel error to $E_{\mathrm{protocol}}=e/2$ in the two-basis version and $E_{\mathrm{protocol}}=e/3$ in the three-basis configuration. This improvement effectively doubles the tolerable QBER threshold relative to BB84 (up to 22%), enabling reliable operation in noisy or lossy optical channels. Because the protocol remains conceptually equivalent to BB84 up to the sifting phase, it inherits its well-established theoretical security while simplifying experimental implementation.

Security is reinforced through multiple layers of physical and statistical defense. Continuous round-trip monitoring enables real-time detection of man-in-the-middle (MiTM) attempts via delay-invariance analysis, while the passive nature of Bob’s module inherently mitigates photon-number-splitting (PNS) and side-channel attacks.

Overall, the Loop-Back QKD protocol constitutes a practical and theoretically grounded alternative to classical authentication and key-exchange methods, such as QR-based or NFC-based secure identification. Its lightweight optical architecture, coarse synchronization tolerance, and resilience to depolarization make it an appealing candidate for integration into emerging quantum-mobile ecosystems. While current results are primarily theoretical, ongoing experimental validation aims to confirm the feasibility, scalability, and robustness of this protocol in real-world mobile and distributed quantum communication scenarios.

## Figures and Tables

**Table 1:** Summary of the main error models and security considerations addressed for the Loop-Back QKD protocol.

Aspect	Model/Scenario	Implication for Loop-Back QKD
Channel depolarization	Isotropic depolarizing channel with error probability *e* per one-way use	Depolarization is mapped into an effective QBER E=e/2 (n=2) or E=e/3 (n=3). The resulting thresholds $e_{\mathrm{th}}^{\left(2\right)}\approx 0.22$ and $e_{\mathrm{th}}^{\left(3\right)}\approx 0.48$ quantify the enhanced tolerance to symmetric polarization noise with respect to one-way DV-QKD.
Photon-number effects	Weak coherent pulses with Poissonian statistics and PNS-type strategies	Loop-Back shares the usual vulnerabilities of WCP-based DV-QKD to photon-number-splitting attacks. Standard decoy-state methods can be incorporated, as in BB84, to bound Eve’s information on multi-photon components; this aspect is not fundamentally modified by the Loop-Back geometry.
Side channels at the remote node	Power/timing leakage and bright-light or Trojan-horse probes on Bob’s module	By removing single-photon detectors and key-dependent post-processing from Bob, Loop-Back suppresses dominant detector side channels at the remote node. The EOM operated at constant low power reduces timing leakage, while standard countermeasures such as optical isolation and power monitoring remain necessary against bright-light and Trojan-horse attacks.
Implementation scope	Misalignment, dark counts, timing jitter and finite-size statistics	Practical imperfections are treated as additional contributions to the QBER on top of the depolarizing model and are expected to affect Loop-Back and one-way DV-QKD in a comparable way for similar hardware. The reported error thresholds are therefore asymptotic benchmarks under symmetric collective attacks; a full finite-size and device-level analysis is left for future work.

## Data Availability

Data is contained within the article.

## Appendix A. Branch Analysis of the MiTM Attack

In this appendix we list the branch probabilities for the representative case in which Alice sends $|0_{Z}\rangle$ and Eve measures in the *X* basis, obtaining the forward outcome $|0_{X}\rangle$. The structure is symmetric for the other inputs and outcomes discussed in Section 6. Table A1 summarizes these branches for the MiTM attack considered in the security analysis.

**Table A1:** Branch probabilities for the MiTM attack when Alice sends $|0_{Z}\rangle$ and Eve measures in the *X* basis, obtaining the forward outcome $|0_{X}\rangle$. The sum of all branch probabilities is 1, and the sum of the rows in which Alice obtains $|1_{Z}\rangle$ yields $p_{⊥}^{\left(X/Y\right)}=5/6$, consistently with the analysis in Section 6.

Bob Basis $B_{B}$	Bob Operation	Eve (Second)	Alice (*Z* Outcome)	Probability
*X*	0X	0X	$0_{Z}$	$\frac{1}{3}\cdot \frac{1}{2}$
		0X	$1_{Z}$	$\frac{1}{3}\cdot \frac{1}{2}$
*Y*	0Y	0X	$0_{Z}$	$\frac{1}{3}\cdot \frac{1}{2}\cdot \frac{1}{2}$
		0X	$1_{Z}$	$\frac{1}{3}\cdot \frac{1}{2}\cdot \frac{1}{2}$
		1X	$0_{Z}$	$\frac{1}{3}\cdot \frac{1}{2}\cdot \frac{1}{2}$
		1X	$1_{Z}$	$\frac{1}{3}\cdot \frac{1}{2}\cdot \frac{1}{2}$
*Y*	1Y	0X	$0_{Z}$	$\frac{1}{3}\cdot \frac{1}{2}\cdot \frac{1}{2}$
		0X	$1_{Z}$	$\frac{1}{3}\cdot \frac{1}{2}\cdot \frac{1}{2}$
		1X	$0_{Z}$	$\frac{1}{3}\cdot \frac{1}{2}\cdot \frac{1}{2}$
		1X	$1_{Z}$	$\frac{1}{3}\cdot \frac{1}{2}\cdot \frac{1}{2}$
*Z*	0Z	0X	$0_{Z}$	$\frac{1}{3}\cdot \frac{1}{2}\cdot \frac{1}{2}$
		0X	$1_{Z}$	$\frac{1}{3}\cdot \frac{1}{2}\cdot \frac{1}{2}$
		1X	$0_{Z}$	$\frac{1}{3}\cdot \frac{1}{2}\cdot \frac{1}{2}$
		1X	$1_{Z}$	$\frac{1}{3}\cdot \frac{1}{2}\cdot \frac{1}{2}$
*Z*	1Z	0X	$0_{Z}$	$\frac{1}{3}\cdot \frac{1}{2}\cdot \frac{1}{2}$
		0X	$1_{Z}$	$\frac{1}{3}\cdot \frac{1}{2}\cdot \frac{1}{2}$
		1X	$0_{Z}$	$\frac{1}{3}\cdot \frac{1}{2}\cdot \frac{1}{2}$
		1X	$1_{Z}$	$\frac{1}{3}\cdot \frac{1}{2}\cdot \frac{1}{2}$
